# Association Mapping for Biomass and Kernel Traits in Doubled-Haploid Population Derived from Texas Wheat Cultivars

**DOI:** 10.3390/genes16101172

**Published:** 2025-10-05

**Authors:** Yahya Rauf, Zhen Wang, Kyle Parker, Shannon A. Baker, Jason A. Baker, Jackie C. Rudd, Qingwu Xue, Amir Ibrahim, Shuyu Liu

**Affiliations:** 1 Texas A&M AgriLife Research and Extension Center, 6500 W Amarillo Blvd, Amarillo, TX 79106, USA; yahya.rauf@ag.tamu.edu (Y.R.);; 2 Department of Soil and Crop Science, Texas A&M University, College Station, TX 77843, USA; 3 Texas A&M AgriLife Research, 600 John Kimbrough Blvd, College Station, TX 77843, USA

**Keywords:** association mapping, QTL, kernel traits, biomass, winter wheat, doubled haploids, image analysis, Illumina NovaSeq

## Abstract

Background: Genetic improvement in wheat yield is the most focused research area for the breeding community to ensure sustainable production. Wheat kernel traits and biomass are considered key contributors to enhance crop yield. Methods: This study was designed to explore the genetic diversity of kernel and biomass traits in popular wheat varieties from the US Southern Great Plains using 264 doubled haploid (DH) lines mainly derived from TAM 114 or TAM 204. This population was evaluated in two field environments planted in alpha lattice design during the 2020 crop season. Kernel traits were collected using the hp Scanjet G4010 photo scanner for image capturing and GrainScan v3. software for image analysis. Biomass parameters were collected and processed manually. For genotyping genomic libraries were prepared and sequenced on Illumina NovaSeq 6000 to generate paired end reads of 150 bp. Sequences were aligned to the IWGSC RefSeq genome assembly v2.1 using the Burrows Wheeler Aligner for SNP calling. Results: A total of 59,482 polymorphic SNP markers were retained for genetic analysis after the filtration at 50% missing data and 5% minor allele frequency. To investigate the marker–trait association and the genomic regions, four genome-wide association study models were implemented using the *R* package GAPIT version 3.5. Based on the Bonferroni correction <8.41 × 10^−7^ was used as a threshold to declare marker-trait associations (MTAs) significant. The BLINK model identified 12 MTAs on chromosomes 1A, 2A, 2B, 4A, 4B, and 6B. Conclusions: The identified MTAs can be used to develop diagnostic markers for efficient selection and utilization in recombination breeding and cultivar development process.

## 1. Introduction

The bread wheat (*Triticum aestivum* L.) feeds almost 30% of the world population and is a major source of up to 25% daily calories consumed by humans globally [1]. Rapid population growth, climate changes, and frequent global events of biotic and abiotic stress are big agricultural challenges for sustainable production. The wheat yield needs to be increased 2.5% annually and under current circumstances it is projected that by 2050 overall wheat production should increase up to 70% to meet the future demand [2,3,4]. The kernel traits and biomass are very critical elements contributing directly to yield and yield components in wheat and have great potential in exploiting the genetic diversity within the adopted cultivars, conventional germplasm, and Triticeae gene pool [5]. Measuring kernel traits is a very crucial component in cereal breeding and genetics and high phenotypic accuracies provide more reliable genetic insights. Manual wheat kernel phenotyping is very laborious, time consuming, and expensive, while the GrainScan approach is a high-throughput, robust, and cost-effective phenotyping platform [6,7].

Traits contributing to the yield and yield components mainly exhibit polygenic inheritance and controlled by several quantitative trait loci (QTL) and genes [8]. Genome-wide association studies (GWAS) and linkage mapping have been widely used to elucidate the genetic mechanisms of complex traits [9,10]. Through several studies, the effectiveness of GWAS has been proven to identify the marker–trait association for agronomic traits [11], disease resistance [12], end-use quality [13] and yield related traits [14]. In wheat, QTL and genes for spikes [15] and kernel traits have been reported on all chromosomes [16,17]. Few major effect genes and QTL, especially *TaGS5*, *TaSus1*, *TaSus2*, and *TaGW2*, are involved in genetically controlling kernel size, kernel weight, spike, peduncle length, and grain weight in wheat [18,19,20]. The major genes *Q* & *C* on chromosomes 5A and 2D, controlling the modern wheat spike morphology are also associated with kernel size, shape, grain yield, and 1000 kernel weight [21,22].

Numerous studies have been conducted to investigate the genetic mechanisms controlling the kernel and biomass traits in spring and winter wheat germplasms, but continuous efforts to identify new sources of diversity are still a key to success in developing improved cultivars. The popular cultivated winter wheat varieties in the US Southern Great Plains likely harbor unexplored allelic diversity and potentially new QTL contributing to the kernel traits and overall, to the yield. The wheat genetics program at the Texas A&M AgriLife Research in Amarillo, TX developed an association-mapping panel involving the most popular cultivated winter wheat cultivars in the region using the doubled haploid (DH) approach. This approach has been successfully used to pyramid favorable alleles from different sources by reducing the breeding cycles, attaining homozygosity in a short period of time and accelerating the genetic gain in wheat [23].

This study used a winter wheat DH association-mapping panel that was phenotyped for kernel and biomass traits using the high-throughput seed scanner and genotyped on the NovaSeq 6000 next-generation sequencing platform. We performed GWAS with the objectives to identify genomic regions associated with biomass and kernel traits by integrating the cost-effective and efficient seed-scanning approach and exploring the underlying genetics.

## 2. Materials and Methods

### 2.1. Association-Mapping Panel

The association-mapping panel comprised 264 doubled haploid (DH) lines mainly derived from the cultivated winter wheat varieties ‘TAM 114’ and ‘TAM 204’ in Southern Great Plains. The other parents are widely adopted and improved cultivars from the region. A complete list of lines involved in DH population development are listed in Table S1. TAM 114 is a hard red winter wheat which was developed and released by the Texas A&M AgriLife Research in 2014. It has awns and red glumes with medium maturity and semi-dwarf features. This variety was released due to the distinguishing characteristics including very strong baking properties, high mixing tolerance, and excellent loaf volume. TAM 114 stands out as a high yielding cultivar in both irrigated and dryland environments of Texas High Plains as compared to the famous TAM 111 and TAM 112 varieties. It has high test weight, exhibited resistance to all three rusts (leaf, stripe, and stem) at the time it was released, with a moderate resistance to Hessian fly biotypes GP and vH9. TAM 204 is also a high-yielding, drought-tolerant variety cultivated in the Texas Great Plains. It is an awnless variety with red glumes and is mainly used for grazing. It also provides resistance against wheat streak mosaic virus (WSMV), greenbug (GB), Hessian fly (HF), and wheat curl mite (WCM).

#### Development of Doubled Haploids (DH)

The wheat genetics lab at the AgriLife Research in Amarillo, TX developed doubled-haploids by pollinating the F_1_ plants with the corn pollen and treating pollinated spikes with 2,4-Dichlorophenoxyacetic acid (2,4-D) synthetic auxin solution (a plant-growth hormone) to induce haploid embryo formation. Two weeks after the pollination, embryos were rescued by dissecting seeds, treated with colchicine solution for chromosome doubling, incubated for 16–24 h at 18 °C and then cultured on the Murashige–Skoog (MS) growth media for 7 days in a dark room. After germination seedlings were transferred to the growth chamber until two leaf stage and then vernalized at 4 °C for 6–8 weeks. The vernalized seedlings were transplanted into 2.5-inch pots filled with potting mix and transferred into growth room to recover for 1–2 weeks. Later, the plants were moved to greenhouses until the physiological maturity. Each plant was assigned a unique identification number based on parentage during the harvest to develop a population for downstream genetic studies.

### 2.2. Kernel Phenotyping, Biomass and Statistical Analysis

#### 2.2.1. Experimental Layouts

A set of 264 DH lines was planted in two environments; Bushland irrigated and Bushland dryland (35°06′ N, 102°27′ W) in the year 2020, hereafter designated as BI20 and BD20. Both environments are very consistent over the years, and we used field data from only one season, which was a limitation in capturing year-to-year environmental variation. An alpha-lattice experimental design was implemented in both environments with a plot size of 3.05 m long and 1.52 m wide for the irrigated and 4.57 m by 1.52 m for the dryland experiments. A total of seven rows were planted for each plot with rows 20 cm apart and plants spaced at 10 cm for irrigated and 15 cm for dryland experiments. The experimental layout used incomplete blocks with one replication, and treatments (DH lines) were randomly assigned to the blocks. Both experiments included four check varieties, TAM 114, TAM 115, TAM 204, TAM 205 in the experimental layout.

#### 2.2.2. Kernel Image Capturing

**Figure 1:**
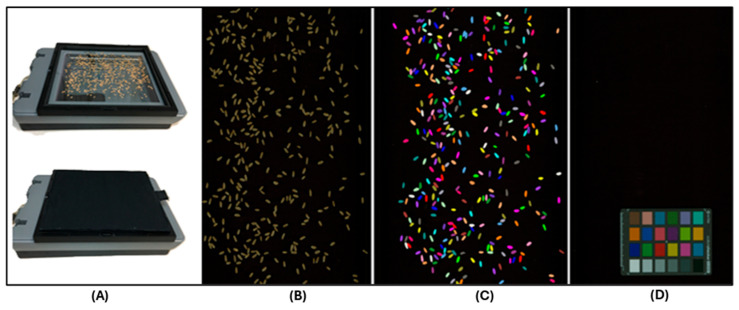
Wheat seed scanning and image processing; a high-throughput, robust, and cost-effective seed trait-phenotyping approach. (**A**) Scanjet G4010 photo scanner (*Hp* 11956A, Hewlett-Packard, Palo Alto, CA, USA) with black cardboard placed over the scanning surface to minimize reflection and shadow. (**B**) Wheat seeds scattered on the flat screen, avoiding seed contacts at pre-image capturing stage. (**C**) Post-seed scanning output image with color calibration and each colored dot represents a single data point for downstream image analysis. (**D**) Munsell ColorChecker Mini card used for standardization of color measurements to the CIELAB colorspace.

Approximately 7 to 8 g seeds of each DH line from both environments (BI20 and BD20) were used for kernel traits phenotyping using the *hp* Scanjet G4010 photo scanner (Hp 11956A, Hewlett-Packard, Palo Alto, CA, USA) which is a consumer-grade flatbed scanner (Figure 1A). All images were scanned at 300 dots per inch (DPI) with no color adjustment or cropping applied. The DPI measures the density of dots in an image and describes the resolution of a digital display. For wheat scanning, grain samples from each DH line were spread out in a glass-bottomed tray (Figure 1B). To counter and mitigate any shadow or reflections, black cardboard was placed over the seed-scanning surface. To allow for the standardization of color measurements to the CIELAB colorspace, a Munsell ColorChecker Mini card (X-Rite Corp., Grand Rapids, MI. USA) was scanned using the same parameters which were later used for seed scanning to generate conversion parameters for the color information (Figure 1C,D). We used an equal amount of seed (7 to 8 g) from each line to avoid excessive touching of grains during the scanning process and ensure separate data point generation from each seed. The total seed count per image ranged from 201 to 483 with a mean value of 318 in the BD20 environment, while it ranged from 167 to 389 with a mean value of 264 in BI20 environment.

#### 2.2.3. Image Analysis and Data Generation

In GrainScan, image analysis used a grayscale image derived from the scanned color image by averaging the red and green channels. We utilized preprocessing that simplified the image, the factors which are used in the simplification process are mainly interconnected components [24]. This preprocessing involves the Gaussian smoothing that minimizes noise, based on an attribute on width (*0.3 × Min grain width*) to fill in the grain crease, a thinning attribute based on elongation to remove background scratches, and attributes based on width (*0.7 × Min grain width*) and length (*0.7 × Min grain length*) to remove thin and thick debris, respectively. The Gaussian smoothing or Gaussian blur calculates the average of neighboring values to effectively blur or smooth an image which are based on the computer algorithms. The following formula is used for a 2D Gaussian function to process an image:

G (x, y) = (1/(2π σ^2^)) × e^ (−(x^2^ + y^2^)/(2σ^2^))

where

G (x, y): Gaussian value at (x, y) coordinates.
σ (sigma): Standard deviation, controlling the width or blurriness of the Gaussian.
x and y: Horizontal and vertical distances from the center of the Gaussian kernel.
e: Base of the natural logarithm.
π: Mathematical constant (Pi).

The (1/ (2π σ^2^)) term is a normalization factor that ensures the integral of the Gaussian function over all space is equal to 1. This is important for maintaining the overall brightness of the image.

During the image processing, a common global threshold is utilized to separate grains from the background implementing an automated method. The method is based on a bivariate histogram of input gray versus gradient. This is a very reliable procedure and commonly used in image normalization [25]. A binary object-splitting method is used to separate the grains if they are very close and touch each other. To remove any small regions created by the grain splitting step, a filtering based on the connected component area (*0.5 × Min grain width × Min grain length*) is performed. Individual grains are labeled and measured based on their size and color. The dimensional measurements are area, perimeter, length, and width with the major and minor axes of the best fit ellipse (called majellipse and minellipse, respectively). Moreover, the kernel image dimension units are converted from pixels to millimeters (mm) based on the input scanner resolution in DPI.

The GrainScan software has two independent procedures while performing color analysis. The first procedure calculates the color measurements for individual grain as CIELAB values rather than the raw RGB values. To use the color calibration option, the image of a calibrated color checker card must first be analyzed using ColourCalibration software. This software performs following functions:

(i) Locating the card; (ii) segmenting the color swatches; (iii) extracting of RGB mean values for each swatch; and (iv) calculate transformation matrix (RGB2Lab) through a linear regression of measured RGB values and the supplied CIELAB values for each swatch.

Using the transformation matrix within GrainScan v3. software, the colored measurements made within each labeled grain can be converted from raw RGB values to calibrated L*, a*, and b* values. CIELAB expresses color as three values: L* for perceptual lightness and a* and b* for the four unique colors of human vision; red, green, blue, and yellow.

The GrainScan v3. software [26] calculated the kernel area (area, mm^−2^), kernel perimeter (peri, mm), kernel length (length, mm), and kernel width (width, mm) for each individual seed from each DH line. We developed the *R* script to compile those varying individual data points to obtain an average trait value for each line. The average trait value was used for genetic analysis. Based on the weight of the scanned seed (e.g., 7 g) and numbers of seeds (e.g., 312 seeds) from each DH line, we calculate the seed weight kernel^−1^ (7/312 = 0.022 g) and 1000 kernel weight (0.022 × 1000 = 22 g).

#### 2.2.4. Biomass Traits

The following above ground biomass traits were recorded on all DH lines in both environments.

- Plant weight (P. Wt, g): A half-meter-long single row representing the plot was harvested from the 2nd or 3rd row of the plot at the time of physiological maturity, and the weight was measured including the stem, leaves, and heads.
- Head count (H. Count): The number of heads were counted and recorded from the same plant.
- Head weight (H. Wt, g): Heads were separated from the stems at the base of the spike and all heads were weighed together from the same plant.

#### 2.2.5. Data Analysis

The Pearson correlation coefficients (*r*) were calculated for all eight traits in both phenotyping environments in ‘*R* Studio’ using the ‘metan’ package. The basic statistics describing data quality for both data sets were performed in *IBM SPSS1.0.0.1174* (IBM Corp., Armonk, NY, USA).

### 2.3. Genotyping and SNP Calling

The 264 DH lines were planted in a 126-well plastic tray filled with cotton balls soaked with distilled water. Trays were placed in a dark room for 48 h for seed germination and later shifted to a growth chamber at 18 °C for a 12 h day length. When plants had grown to the two-leaf stage, approximately 2 cm leaf tissue was harvested, placed in 2 mL microtubes, and lyophilized for 3 days. The leaf tissues were ground using the Plant TissueLyzer II (QIAGEN) and DNA extraction was performed on the BioSprint workstation (QIAGEN) using the BioSprint 96 DNA plant kit [27]. Genomic libraries were prepared using the TrueSeq DNA PCR-Free kit. This kit is specifically designed for the whole-genome sequencing and better coverage for complex genomes. Libraries were denatured and diluted and sequenced on NovaSeq 6000. Demultiplexing was performed using the bioinformatics pipeline to sort reads and separate them by their unique barcodes [28]. For SNP identification, the IWGSC Chinese Spring wheat genome assembly v2.0 was used for sequence alignment. The ALN function in Burrows–Wheeler Aligner with default parameters was implemented to align the sequences with the reference genome [29]. Samtools further processed the aligned sequences, and the “mpileup” procedure along with bcftools were used for SNP calling [30]. The final SNP filtration was performed at 50% missing data and 5% minor allele frequency [31]. SNP filtration threshold is a trade-off between data retention and data quality. Many genotyping platforms including the genotyping-by-sequence (GBS) generate a high volume of incomplete data and under these circumstances 50% SNP filtration cutoff is justifiable to avoid purging a large number of markers. We used Beagel 5.0 for the genotype imputation, which implements the Hidden Markov Model [32]. Genetic data was imputed based on how two individuals are identical-by-descent (IDB). Our SNP calling and SNP filtration workflow pipeline was as below:

Raw SNP file with 50% missing data -> set Het = NA -> refilter for 50% missing -> impute missing calls -> filter for MAF = 0.05 and H = 0.1.

### 2.4. Association-Mapping Analysis

To identify the marker–trait associations and the genomic regions, a genome-wide-association study (GWAS) analysis was conducted using the *R* package ‘Genome Association and Prediction Integrated Tool’ (GAPIT) [33]. We implemented four different models called Mixed Linear Model (MLM) [34], Multiple-Locus Mixed Linear Model (MLMM) [35], Fixed and random model Circulating Probability Unification (FarmCPU) [36], and Bayesian information and Linkage-disequilibrium Iteratively Nested Keyway (BLINK) [37]. Each model has its own advantages and disadvantages, but the MLM can simply be described using Henderson’s matrix notation as follows:

*Y* = *Xβ* + *Zu* + *e*

In this matrix notation, *Y* is the vector for phenotypes; *β* is an unknown vector containing fixed effects, which can include genetic markers, population structure (Q), and an intercept; *u* is an unknown vector of random additive genetic effects from multiple background QTL for individuals/lines; *X* and *Z* are the known design matrices; and *e* is the unobserved vector of residuals. The *u* and *e* vectors are assumed to be normally distributed with a null mean and a variance of:

$$var=\left[\begin{array}{c} u\\ e \end{array}\right]=\left[\begin{array}{cc} G & 0\\ 0 & R \end{array}\right]$$

where *G* = *σ*^2^ *_a_K* with *σ*^2^*_a_* as the additive genetic variance and *K* as the kinship matrix. Homogeneous variance is assumed for the residual effect, i.e., *R* = *σ*^2^*_e_**^I^***, where *σ*^2^*_e_* is the residual variance. The proportion of the total variance explained by the genetic variance is defined as heritability (*h*^2^).

(1) $$h^{2}=\frac{\sigma_{a}^{2}}{\sigma_{a}^{2}+\sigma_{e}^{2}}$$

In GAPIT, the covariate variables include the first three principal components derived from all the markers and the origin group. Principal component analysis was performed using all available SNPs. The first principal components were fitted as covariate variables to reduce the false positives due to population stratification. The portion of variance explained by each component was as follows:

(2) $$\frac{Portion\;explained\;by\;each\;PC}{Total\;variance}$$

where the total variance was sum of all the eigenvalues of the available SNP data set.

In the MLM, DH lines were considered as a random effect and the relevance among them was derived by a kinship matrix. The elements in the matrix were utilized as similarities and the resultant clusters were visualized using an Unweighted Pair Group Method with Arithmetic Mean (UPGMA) based heatmap in GAPIT package. We used the Bonferroni correction to declare significant marker–trait association. This method adjusts the significance level (alpha) to control the family-wise error rate (FWER), which is the probability of making at least one false-positive call in the entire analysis. The adjusted significance level (α`) was calculated as follows:

(3) $$\alpha `=\;\frac{alpha}{n}$$

where alpha is 0.05 and *n* is the number of tested markers.

## 3. Results

### 3.1. Phenotyping and Statistical Analysis

The seed-scanning process resulted in an average of 483 and 264 data points from each DH line in BD20 and BI20 phenotyping environments, respectively. These results were expected from both experiments because in BD20 environment 7–8 g seeds counted a higher number due to stressed growing conditions producing shriveled seeds, while the BI20 environment has optimal growing conditions with healthy seeds and 7–8 g counted a smaller number of seeds. The phenotypic data for all traits in both environments had continuous distribution spectrum in the DH association-mapping panel (Tables S1 and S2). The basic statistical measures for all the eight traits in both phenotyping environment reflected the data quality and phenotyping accuracies. The standard deviations which quantify the spread of individual data points around the sample means were low for all traits in both environments. One of the major yield components ‘1000 KW’ ranged 19.9 to 43.7 (x̄ = 29.7; σ = 4.4) and 15.8 to 36.0 (x̄ = 23.8; σ = 3.6) in BI20 and BD20 environments, respectively. The standard errors measured the precision of the sample means as an estimation of the population mean which ranged from 0.0 to 1.4 and 0.0 to 1.3 in BI20 and BD20 environments, respectively (Tables S3 and S4).

**Figure 2:**
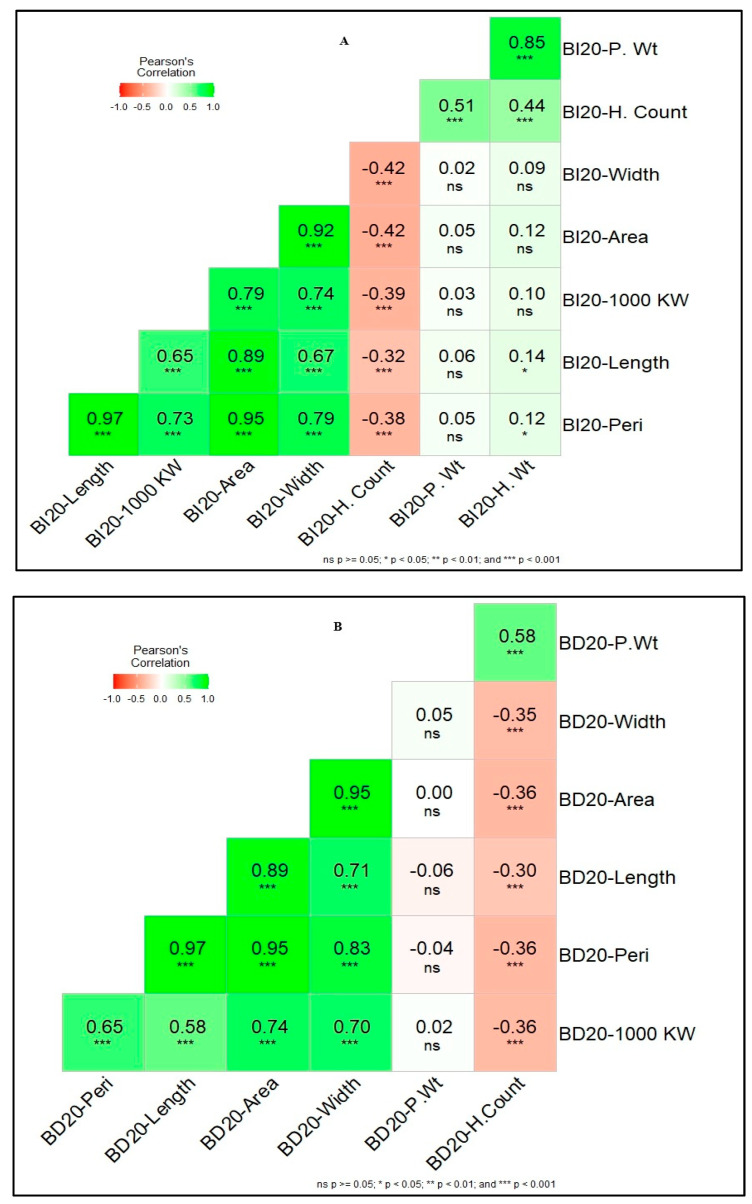
Pearson correlation coefficient (*r*) among all traits in both phenotyping environments. In (**A**); BI20 represents Bushland irrigated and in (**B**); BD20 represents Bushland dryland environments in 2020 crop cycle. The correlations with *p* ≥0.05 are non-significant represented as ‘ns’, while the correlations with *p* < 0.05, 0.01, and 0.001 are significant and represented by asterisks (*, **, ***), respectively. The legend bar scale differentiates the significant (dark green) and non-significant (dark red) correlation.

Pearson coefficient correlations (*r*) were highly significant for length, 1000 KW, area and width (*r* = 0.65 to 0.97) at *p* < 0.001, while non-significant for P. Wt and H.Wt with Kernel traits in BI20 environment. The H. Count trait was negatively significantly correlated with all the kernel traits (*r* = −0.32 to −0.43, *p* < 0.001) (Figure 2A). For the BD20 environment, almost similar correlation trends were observed for the kernel traits which had highly positive significant correlation (*r* = 0.58 to 0.97) at *p* < 0.001. The biomass traits, H.Count were negatively correlated with kernel traits including kernel weight (*r* = −0.30 to −0.36) at *p* < 0.001. In both BD20 and BI20, P.Wt and H.Count were highly correlated (r = 0.51 to 0.58, *p* < 0.001) but P.Wt was not significantly correlated with all kernel traits (Figure 2A,B).

### 3.2. Genomic Libraries and SNP Calling

The genomic libraries sequenced on NovaSeq 6000 to generate 150 bp double-ended reads with an average 1.3–1.6 billion reads per sample. Genomic libraries passed the pre- and post-size selection quality control analysis for both size and mass (Agilent size = 50–650 bp; pooled sample concentration = 7 to 11 nM). Approximately 95% of the reads generated passed the Q30 quality criteria with a mean read quality score of 36. The SNP calling workflow provided 3,702,918 raw variants at minimum loci coverage of 50 (MLC50), which also represents that 50% variant calls were missing. We performed filtering using a criterion of Het = 0.2 and MLC50, which purged 1,783,410 SNP retaining 1,919, 508 SNP. After the SNP imputation another data filtration was performed at MAF = 0.05 and Het = 0.1 purging 1,860,026 SNP and finally 59,482 markers were retained for the GWAS analysis (Table S5).

The total physical distance/length of the genome was 14,073.32 Mb with an average 0.236 Mb whole-genome marker density. The B genome had a maximum number of polymorphic SNP markers (26,366) followed by A genome (22,710) and D genome (10,406). Chromosome 3B had the highest number of markers (8322), while chromosome 4D had the least number of markers (1079) (Table 1).

### 3.3. Population Structure, Maker Heterozygosity and Kinship Matrix

**Figure 3:**
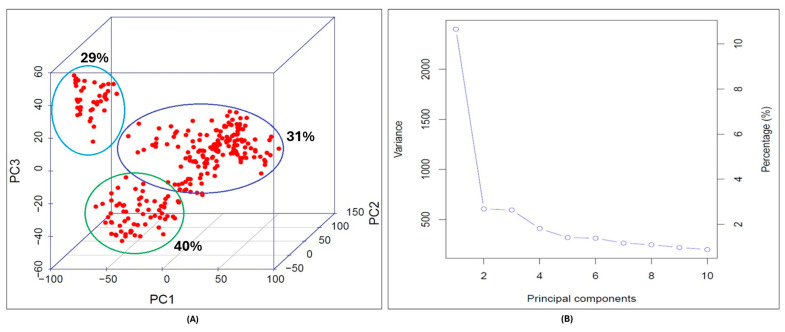

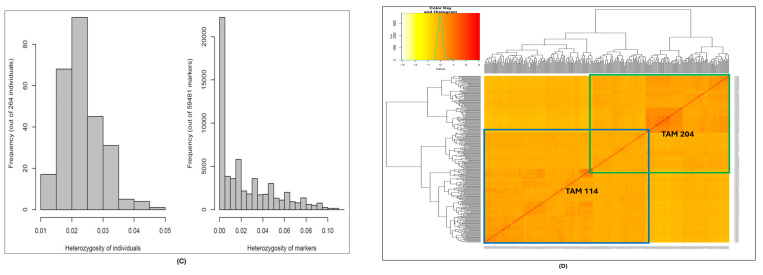
Population structure analysis of DH wheat association-mapping panel. (**A**) Principal component analysis (PCA) showing three sub-populations in the mapping panel. (**B**) The percentage of the variance explained by the principal components. (**C**) Frequency of heterozygous DH lines in the association-mapping panel and heterozygous markers. (**D**) Heat map of kinship matrix representing relatedness among the population. The darker regions show higher co-efficient co-ancestry between genotypes and dendrograms depicts clustering of sub-populations within the panel.

The principal component analysis (PCA) captured the most significant genetic variation within the association-mapping panel. The data visualization identified three distinct clusters representing the sub-populations (Figure 3A). The PCA in conjugation with EIGENSTRAT accounted for the population stratification and eigenvectors associated with each PCA showed that the maximum percentage of variation was explained by PC1 followed by PC2, and PC3 accounted for the least variation in the population. The PCA calculated covariance matrix of the data to output its eigenvalues and eigenvectors. Each eigenvalue was associated with an eigenvector also called component vector (Figure 3B). The total variance was 2.05, based on the sum of all the eigenvalues in the SNP data. Among the principal components (PC1, PC2, PC3) the eigenvalues were λ1 = 0.80, λ2 = 0.64, and λ3 = 0.60 for each component, respectively. The PC1, PC2, and PC3 explained 40%, 31%, and 29% variation, respectively. (Figure 3A, Table S6).

We observed very low heterozygous frequency for both the SNP makers and the DH lines in the population (Figure 3C). The kinship matrix represented the relatedness among individuals with the population and dendrograms depicted clustering of sub-populations within the panel. The dark regions in the heat map show higher co-efficient co-ancestry between individual genotypes and three sub-populations clearly distinguished in the kinship matrix (Figure 3D). Through a closer look at the kinship matrix and the DH line’s pedigree list we were able to distinguish between two large rectangles. The upper right rectangle (Figure 3D; green bordered) in the heat map represents the TAM 204 derived lines, while the lower left rectangle (Figure 3D; blue bordered) represented the TAM 114 derived lines. Furthermore, we also observed subgroups within these two major groups which represented some other lines from the Southern Great Plains that have either TAM 114 or TAM 204 blood in their background. The blue and green rectangles overlap each other in the middle of the heatmap, those lines shared the pedigree from both TAM 114 and TAM 204 varieties.

### 3.4. Genome Wide Association Mapping

For this manuscript, the GWAS results presented are based on the BLINK model due to its computing efficiency and statistical power. The GWAS analysis in GAPIT using the BLINK model identified 12 marker–trait associations (MTAs) on chromosomes 1A, 2A, 2B, 4A, 4B, and 6B (Table 2). The threshold level to declare a MTA significant was based on the Bonferroni correction. We calculated the adjusted alpha based on the significant *p*-value = 0.05 and 59,482 SNP markers which resulted in a *p* < 8.41 × 10^−7^ to declare any MTA significant. The LOD values for each significant MTA were based on the *-base10 log* of the actual *p*-value.

## 4. Discussion

Grain yield in wheat is a highly desirable trait which is mainly polygenic with quantitative inheritance and influenced by the genetic background and environmental factors [38]. There are two essential components, 1000 KW and number of grains m^−2^ contributing directly to grain yield. These components have significantly impacted the yield over the wheat-breeding history [39]. In the current study, we focused on kernel traits, including length, width, area, and peri, which determine the 1000 KW. This agronomic trait is very stable, and breeders make selection based on 1000 KW during the variety development process [40]. It is evident from previous studies that 1000 KW and other kernel traits have higher contribution to the grain yield as compared to number of grains per spike [41]. This study utilized a set of 264 DH lines developed by the Texas A&M AgriLife Research Wheat Genetics Program and were mainly derived from the popular cultivated wheat varieties form the Southern Great Plains.

The phenotyping data from both environments reflected significant variation for all 8 traits and data distribution was continuous which indicates that traits have polygenic and quantitative inheritance. Measured traits had lower standard deviation which illustrates that data points were clustered closer to trait means values in both environments. Similarly, standard error values for all traits were very small, indicating that the sample means were an accurate representation of the population mean. The kernel traits were highly correlated in both environments with the 1000 KW, especially length, width, area, and peri, which supported the concept that kernel traits contribute to 1000 KW and overall, to the grain yield. Some previous studies have also shown moderate-to-high correlations between 1000 KW and kernel size [42]. It has also been reported that kernel length and width in both durum and bread wheat positively influences 1000 KW [43,44]. We observed a consistent non-significant and negative correlation among biomass and kernel traits in both environments. These negative associations could be explained by the environmental factors contributing to plant growth and seed development (Figure 2 A, B).

**Figure 4:**
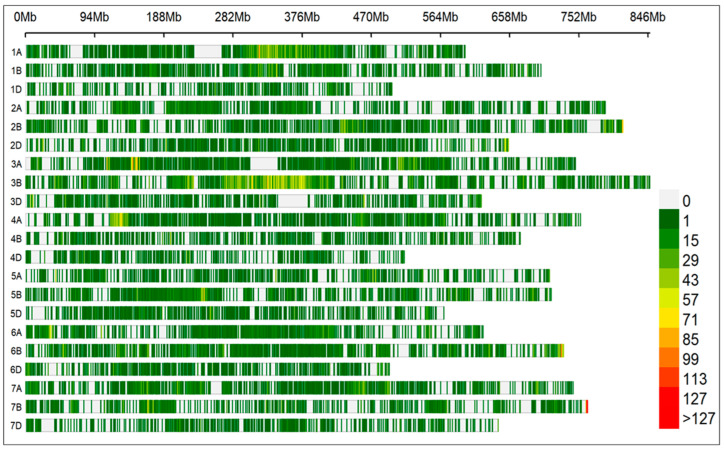
SNP marker distribution and SNP density across 21 wheat chromosomes. The vertical lines of different colors represent the SNP density within 5 Mb window size.

All 21 chromosomes had good coverage and high marker density. The D genome had overall low marker density which is very common in wheat due to less historical recombination events (Figure 4). A total of 59481 SNP markers were deployed to analyze the population structure and the relatedness among 264 DH lines. The PCA distinguished three sub-populations in the mapping panel. Based on the mathematical calculations of total variance and the phenotypic variance explained by each principal component, we were able to verify the genetic variation contributed mainly by three subgroups (Table S6). The frequencies of heterozygous markers and DH lines were low, and such results were obvious and expected, because heterozygous SNP calls were purged during the SNP filtration process and DH lines should have also attained homozygosity during the chromosome doubling process in DH development.

Several GWAS models have been implemented to explore the significant MTAs. We used four models using the GAPIT package in R, but the data presented in this manuscript was derived from the BLINK model based on its stringency and low false discovery rate. Moreover, we adopted quite stringent criteria to declare a MTA significant based on the Bonferroni correction. All MTAs were considered significant if the corrected *p*-value was less than 8.41 × 10^−7^. Several other factors like population, array type, and marker data can influence the threshold level.

In this study, we identified 12 significant MTAs on 6 chromosomes (1A, 2A, 2B, 4A, 4B, and 6B). Chromosome 1A had only one MTA, where a SNP (*S1A_47840044*) at 47.84 Mb region was associated with length. The chromosome 2A had three significant MTAs for 1000 KW (498.73 Mb), length (211.35 Mb), and peri (251.49 Mb), explaining 5.2 to 8.2% phenotypic variance. Chromosome 2B had four significant MTAs for the length (664.43 Mb), peri (702.38 Mb), H. Wt (740.97 Mb), and width (792.75 Mb) which accounted for 4.4 to 37.1% variation. Chromosomes 4A and 6B each had one significant MTA for 1000 KW (663.09 and 567.70 Mb). Chromosome 4B harbored two significant MTAs for area and width at 592.4 Mb. Both these traits were associated with the same SNP marker (*S4B_592421708*) and accounted for 11.7 to 21.6% variation (Table 2). The identified MTA in the DH mapping population is contributing considerably higher variation especially for the kernel traits and 1000 KW and could be a great resource for genetic variability and further utilization in the wheat breeding pipeline to develop high yielding cultivars. The identified MTAs might represent known major genes and QTL previously identified in the same genomic regions, which need further insights to verify their novelty. The identified MTAs can be used to develop diagnostic markers for efficient trait selection, be utilized in the recombination breeding program, and help accelerate the cultivar development process.

## Figures and Tables

**Table 1:** Density and marker distribution across all 21 chromosomes in wheat doubled-haploid mapping population.

Ch	Start Position	End Position	Length (Mb)	No. of Markers	Marker Density
1A	1.95	597.36	595.41	4465	0.133
1B	1.42	700.37	698.95	3094	0.226
1D	2.88	498.05	495.17	1168	0.424
2A	2.4	787.72	785.32	2858	0.275
2B	1.88	812.03	810.15	3581	0.226
2D	2.22	565.44	563.22	2058	0.274
3A	7.23	747.44	740.21	3912	0.189
3B	0.07	848.26	848.19	8322	0.102
3D	2.69	619.37	616.68	1869	0.330
4A	4.02	754.04	750.02	3451	0.217
4B	1.74	672.25	670.51	1567	0.428
4D	0.46	514.92	514.46	1079	0.477
5A	0.85	712.17	711.32	1902	0.374
5B	0.08	714.55	714.47	3297	0.217
5D	1.58	568.63	567.05	1487	0.381
6A	1.54	621.88	620.34	2765	0.224
6B	1.31	731.18	729.87	3980	0.183
6D	0.06	494.6	494.54	1433	0.345
7A	0.23	743.92	743.69	3357	0.222
7B	1.04	763.61	762.57	2525	0.302
7D	1.25	642.43	641.18	1312	0.489
Whole genome			14,073.32	59,482	0.236

**Table 2:** Significant marker–trait associations identified using the BLINK model for eight traits in two environments.

SNP	Position (bp)	Position (Mb)	Alleles	Chr	*p* Value	LOD	MAF	PVE	Environment/Trait
S1A_47840044	47,840,044	47.840044	A/G	1A	3.09 × 10^−8^	7.51	0.05	10.6	BI20. Length
S2A_498737202	498,737,202	498.737202	C/T	2A	7.41 × 10^−10^	9.13	0.42	08.2	BD20.1000 KW
S2A_211351736	211,351,736	211.351736	C/T	2A	4.02 × 10^−8^	7.4	0.06	05.2	BI20. Length
S2A_251496962	251,496,962	251.496962	G/A	2A	6.49 × 10^−8^	7.19	0.45	05.7	BD20. Peri
S2B_664436363	664,436,363	664.436363	G/A	2B	1.86 × 10^−10^	9.73	0.09	37.1	BD20. Length
S2B_702381983	702,381,983	702.381983	G/C	2B	1.13 × 10^−9^	8.95	0.13	04.4	BD20. Peri
S2B_740979562	740,979,562	740.979562	G/A	2B	2.51 × 10^−9^	8.6	0.33	13.8	BI20. H.Wt
S2B_792756832	792,756,832	792.756832	G/A	2B	6.17 × 10^−8^	7.21	0.39	17.3	BD20. Width
S4A_663097002	663,097,002	663.097002	T/G	4A	3.96 × 10^−11^	10.4	0.23	31.8	BD20.1000 KW
S4B_592421708	592,421,708	592.421708	C/T	4B	1.83 × 10^−13^	12.74	0.25	11.7	BD20. Area
S4B_592421708	592,421,708	592.421708	C/T	4B	4.33 × 10^−12^	11.36	0.25	21.6	BD20. Width
S6B_567706088	567,706,088	567.706088	A/G	6B	7.69 × 10^−9^	8.11	0.25	11.2	BD20.1000 KW

SNP: Single-nucleotide polymorphism; alleles: the first letter is the major allele, while the second letter represents a minor allele. Chr: chromosome name; *p*-value: threshold level necessary to declare a marker–trait association significant. Calculated based on the Bonferroni correction. MAF: minor allele frequency; PVE: phenotypic variance explained.

## Data Availability

All the data attached as “Supplementary Data” will be publicly available.

## Supplementary Materials

The following supporting information can be downloaded at: https://www.mdpi.com/article/10.3390/genes16101172/s1, Table S1: Phenotypic data of association-mapping panel from Bushland irrigated (BI) environment in 2020. Table S2: Phenotypic data of association-mapping panel from Bushland dryland (BD) environment in 2020. Table S3: Basic statistics on all eight phenotypic parameters for data quality in Bushland irrigated (BI) environment in 2020. Table S4: Basic statistics on all eight phenotypic parameters for data quality in Bushland dryland (BD) environment in 2020. Table S5: Genotypic data on 264 doubled-haploid lines using NovaSeq 6000. Table S6: Variance explained by each principal component.

## Abbreviations

The following abbreviations are used in this manuscript:

SNP	Single Nucleotide Polymorphism
MAF	Minor Allele Frequency
PVE	Phenotypic Variance Explained
QTL	Quantitative Trail Loci
GWAS	Genome-Wide Association Study
DH	Doubled Haploid
GAPIT	Genome Association and Prediction Integrated Tool
MLM	Mixed Linear Model
MLMM	Multiple-Locus Mixed Linear Model
BLINK	Bayesian information and Linkage-disequilibrium Iteratively Nested Keyway
FarmCPU	Fixed and random model Circulating Probability Unification
DPI	Dots Per Inch
BD	Bushland Dryland
BI	Bushland Irrigated
MTA	Marker–Trait Association
PCA	Principal Component Analysis
UPGMA	Unweighted Pair Group Method with Arithmetic Mean

## References

[1] Rabieyan E., Alipour H. (2021). NGS-based multiplex assay of trait-linked molecular markers revealed the genetic diversity of Iranian bread wheat landraces and cultivars. Crop Pasture Sci..

[2] Li P., Ma B., Palta J., Ding T., Cheng Z., Lv G., Xiong Y. (2021). Wheat breeding highlights drought tolerance while ignores the advantages of drought avoidance: A meta-analysis. Eur. J. Agron..

[3] Tilman D., Balzer C., Hill J., Befort B.L. (2011). Global food demand and the sustainable intensification of agriculture. Proc. Nat. Acad. Sci..

[4] Ray D.K., Mueller N.D., West P.C., Foley J.A. (2013). Yield trends are insufficient to double global crop production by 2050. PLoS ONE.

[5] Mujeeb-Kazi A., Gul A., Ahmad I., Farooq M., Rauf Y., -ur Rahman A., Riaz H. (2009). Genetic resources for some wheat abiotic stress tolerances. Salinity and Water Stress: Improving Crop Efficiency.

[6] Xi Y., Du Y.L., Wang D., Ren J.Y., Luo W.Y., Peng Q., Wang-Ying F., Feng-Ming L. (2024). Wheat genetic progress in biomass allocation and yield components: A global perspective. Field Crops Res..

[7] Ramya P., Chaubal A., Kulkarni K., Gupta L., Kadoo N., Dhaliwal H.S., Chhuneja P., Lagu M., Gupt V. (2010). QTL mapping of 1000-kernel weight, kernel length, and kernel width in bread wheat (*Triticum aestivum* L.). J. Appl. Genet..

[8] Fan M., Zhang X., Nagarajan R., Zhai W., Rauf Y., Jia H., Yan L. (2023). Natural variants and editing events provide insights into routes for spike architecture modification in common wheat. Crop J..

[9] Zhang D., Fan M., Li T., Rauf Y., Liu Y., Zhu X., Jia H., Zhai W., Luzuriaga J.C., Carver B.F. (2025). A natural allele of the transcription factor gene *TaMYB-D7b* is a genetic signature for phosphorus deficiency in wheat. Plant Physiol..

[10] Wu J.Z., Qiao L.Y., Liu Y., Fu B.S., Ragupathi N., Rauf Y., Jia H.Y., Yan L. (2022). Rapid identification and deployment of major genes for flowering time and awn traits in common wheat. Front. Plant Sci..

[11] Sun C., Zhang F., Yan X., Zhang X., Dong Z., Cui D., Chen F. (2017). Genome-wide association study for 13 agronomic traits reveals distribution of superior alleles in bread wheat from the Yellow and Huai Valley of China. Plant Biotechnol. J..

[12] Zhang J., Gill H.S., Halder J., Brar N.K., Ali S., Bernardo A., Amand P.S., Bai G., Turnipseed B., Sehgal S.K. (2022). Multi-locus genome-wide association studies to characterize Fusarium head blight (FHB) resistance in hard winter wheat. Front. Plant Sci..

[13] Chen J., Zhang F., Zhao C., Lv G., Sun C., Pan Y., Guo X., Chen F. (2019). Genome-wide association study of six quality traits reveals the association of the *TaRPP13L1* gene with flour colour in Chinese bread wheat. Plant Biotechnol. J..

[14] Singh K., Saini D.K., Saripalli G., Batra R., Gautam T., Singh R. (2022). WheatQTLdb V2.0: A supplement to the database for wheat QTL. Mol. Breeding.

[15] Gao F., Wen W., Liu J., Rasheed A., Yin G., Xia X., Wu X., He Z. (2015). Genome-wide linkage mapping of QTL for yield components, plant height and yield-related physiological traits in the Chinese wheat cross Zhou 8425B/Chinese Spring. Front. Plant Sci..

[16] Chen G., Zhang H., Deng Z., Wu R., Li D., Wang M., Tian J. (2016). Genome-wide association study for kernel weight-related traits using SNPs in a Chinese winter wheat population. Euphytica.

[17] Jaiswal V., Gahlaut V., Mathur S., Agarwal P., Khandelwal M.K., Khurana J.P., Tyagi A.K., Balyan H.S., Gupta P.K. (2015). Identification of novel SNP in promoter sequence of *TaGW2-6A* associated with grain weight and other agronomic traits in wheat (*Triticum aestivum* L.). PLoS ONE.

[18] Bednarek J., Boulaflous A., Girousse C., Ravel C., Tassy C., Barret P., Bouzidi M.F., Mouzeyar S. (2012). Down-regulation of the *TaGW2* gene by RNA interference results in decreased grain size and weight in wheat. J. Exp. Bot..

[19] Hou J., Jiang Q., Hao C., Wang Y., Zhang H., Zhang X. (2014). Global selection on sucrose synthase haplotypes during a century of wheat breeding. Plant Physiol..

[20] Ma L., Li T., Hao C., Wang Y., Chen X., Zhang X. (2016). *TaGS5-3A*, a grain size gene selected during wheat improvement for larger kernel and yield. Plant Biotechnol. J..

[21] Johnson E.B., Nalam V.J., Zemetra R.S., Riera-Lizarazu O. (2008). Mapping the compactum locus in wheat (*Triticum aestivum* L.) and its relationship to other spike morphology genes of the Triticeae. Euphytica.

[22] Xie Q., Li N., Yang Y., Lv Y., Yao H., Wei R., Sparkes D.L., Ma Z. (2018). Pleiotropic effects of the wheat domestication gene *Q* on yield and grain morphology. Planta.

[23] Niroula R.K., Bimb H.P. (2009). Overview of wheat x maize system of crosses for dihaploid induction in wheat. World Appl. Sci. J..

[24] Salembier Clairon P.J., Wilkinson M. (2009). Connected operators: A review of region-based morphological image processing techniques. IEEE Signal Process. Mag..

[25] Sintorn I.-M., Bischof L., Jackway P., Haggarty S., Buckley M. (2010). Gradient based intensity normalization. J. Microsc..

[26] Whan A.P., Smith A.B., Cavanagh C.R., Ral J.P., Shaw L.M., Howitt C.A., Bischof L. (2014). GrainScan: A low cost, fast method for grain size and colour measurements. Plant Methods.

[27] Rauf Y., Bajgain P., Rouse M.N., Khanzada K.A., Bhavani S., Huerta-Espino J., Singh R.P., Imtiaz M., Anderson J.A. (2022). Molecular characterization of genomic regions for adult plant resistance to stem rust in a spring wheat mapping population. Plant Dis..

[28] Rauf Y., Lan C., Randhawa M., Singh R.P., Huerta-Espino J., Anderson J.A. (2022). Quantitative trait loci mapping reveals the complexity of adult plant resistance to leaf rust in spring wheat ‘Copio’. Crop Sci..

[29] Li H., Durbin R. (2009). Fast and accurate short read alignment with Burrows–Wheeler transform. Bioinformatics.

[30] Li H. (2011). A statistical framework for SNP calling, mutation discovery, association mapping and population genetical parameter estimation from sequencing data. Bioinformatics.

[31] Wang Z., Dhakal S., Cerit M., Wang S., Rauf Y., Yu S., Maulana F., Huang W., Anderson J.D., Ma X.F. (2022). QTL mapping of yield components and kernel traits in wheat cultivars TAM 112 and Duster. Front. Plant Sci..

[32] Browning B.L., Zhou Y., Browning S.R. (2018). A one-penny imputed genome from next generation reference panels. Am. J. Hum. Genet..

[33] Lipka A.E., Tian F., Wang Q., Peiffer J., Li M., Bradbury P.J., Gore A., Buckler E.S., Zhang Z. (2012). GAPIT: Genome association and prediction integrated tool. Bioinformatics.

[34] Yu J., Pressoir G., Briggs W.H., Vroh Bi I., Yamasaki M., Doebley J.F., McMullen M.D., Gaut B.S., Nielsen D.M., Holland J.B. (2006). A unified mixed-model method for association mapping that accounts for multiple levels of relatedness. Nat Genet..

[35] Segura V., Vilhjálmsson B.J., Platt A., Korte A., Seren Ü., Long Q., Nordborg M. (2012). An efficient multi-locus mixed-model approach for genome-wide association studies in structured populations. Nat Genet..

[36] Liu X., Huang M., Fan B., Buckler E.S., Zhang Z. (2016). Iterative usage of fixed and random effect models for powerful and efficient genome-wide association studies. PLoS Genet..

[37] Huang M., Liu X., Zhou Y., Summers R.M., Zhang Z. (2019). BLINK: A package for the next level of genome-wide association studies with both individuals and markers in the millions. Gigascience.

[38] Li T., Deng G., Su Y., Yang Z., Tang Y., Wang J. (2022). Genetic dissection of quantitative trait loci for grain size and weight by high-resolution genetic mapping in bread wheat (*Triticum aestivum* L.). Theor. Appl. Genet..

[39] Kumari S., Jaiswal V., Mishra V.K., Paliwal R., Balyan H.S., Gupta P.K. (2018). QTL mapping for some grain traits in bread wheat (*Triticum aestivum* L.). Physiol. Mol. Biol. Plants.

[40] Duan X., Yu H., Ma W., Sun J., Zhao Y., Yang R. (2020). A major and stable QTL controlling wheat thousand grain weight: Identification, characterization, and CAPS marker development. Mol. Breed..

[41] Ji G., Xu Z., Fan X., Zhou Q., Chen L., Yu Q. (2022). Identification and validation of major QTL for grain size and weight in bread wheat (*Triticum aestivum* L.). Crop J..

[42] Rasheed A., Xia X., Ogbonnaya F., Mahmood T., Zhang Z., Kazi A.M. (2018). Genome-wide association for grain morphology in synthetic hexaploid wheats using digital imaging analysis. BMC Plant Biol..

[43] Simmonds J., Scott P., Brinton J., Mestre T.C., Bush M., Del Blanco A. (2016). A splice acceptor site mutation in *TaGW2-A1* increases thousand grain weight in tetraploid and hexaploid wheat through wider and longer grains. Theor. Appl. Genet..

[44] Halder J., Gill H.S., Zhang J., Altameemi R., Olson E., Turnipseed B., Sehgal S.K. (2023). Genome-wide association analysis of spike and kernel traits in the U.S. hard winter wheat. Plant Genome.

